# SGK-1 protects kidney cells against apoptosis induced by ceramide and TNF-*α*

**DOI:** 10.1038/cddis.2015.232

**Published:** 2015-09-17

**Authors:** D Pastore, D Della-Morte, A Coppola, B Capuani, M F Lombardo, F Pacifici, F Ferrelli, R Arriga, C Mammi, M Federici, A Bellia, N Di Daniele, M Tesauro, G Donadel, D Noto, P Sbraccia, G Sconocchia, D Lauro

**Affiliations:** 1 Laboratory of Molecular Medicine, Department of Systems Medicine, University of Rome Tor Vergata, Rome, Italy; 2 IRCCS San Raffaele Pisana, Rome, Italy; 3 Laboratory of Cardiovascular Endocrinology, Istituto di Ricovero e Cura a Carattere Scientifico (IRCCS), San Raffaele Pisana, Rome, Italy; 4 Department of Medicine, University Hospital ‘Fondazione Policlinico di Tor Vergata', Rome, Italy; 5 Department of Internal Medicine and Biomedical Specialist (DIBIMIS), University of Palermo, Palermo, Italy; 6 Department of Biomedicine, Institute of Translational Pharmacology, National Research Council, Rome, Italy

## Abstract

Ceramide regulates several different cellular responses including mechanisms leading to apoptosis. Serum- and glucocorticoid-inducible protein kinase (SGK)-1 is a serine threonine kinase, which activates survival pathways in response to stress stimuli. Recently, we demonstrated an anti-apoptotic role of SGK-1 in human umbilical endothelial cells treated with high glucose. In the present study, since ceramide induces apoptosis by multiple mechanisms in diabetes and its complication such as nephropathy, we aimed to investigate whether SGK-1 may protect even against apoptosis induced by ceramide in kidney cells. Human embryonic kidney (HEK)-293 cells stable transfected with SGK-1 wild type (SGK-1wt) and its dominant negative gene (SGK-1dn) have been used in this study. Apoptotic stimuli were induced by C_2_-ceramide and TNF-*α* to increase endogenous synthesis of ceramide. Upon activation with these stimuli, SGK-1wt transfected cells have a statistically significant reduction of apoptosis compared with SGK-1dn cells (*P*<0.001). This protection was dependent on activation of caspase-3 and Poly-ADP-ribose-polymerase-1 (PARP-1) cleavage. SGK-1 and AKT-1 two highly homologous kinases differently reacted to ceramide treatment, since SGK-1 increases in response to apoptotic stimulus while AKT-1 decreases. This enhancement of SGK-1 was dependent on p38-mitogen-activated-protein kinases (p38MAPK), cyclic-adenosine-monophosphate/protein kinase A (cAMP/PKA) and phosphoinositide-3-kinase (PI3K) pathways. Especially, by using selective LY294002 inhibitor, we demonstrated that the most involved pathway in the SGK-1 mediated process of protection was PI3K. Treatment with inhibitor of SGK-1 (GSK650394) significantly enhanced TNF-*α*-dependent apoptosis in HEK-293 cells overexpressing SGK-1wt. Caspase-3, -8 and -9 selective inhibitors confirmed that SGK-1 reduced the activation of caspase-dependent apoptosis, probably by both intrinsic and extrinsic pathways. In conclusion, we demonstrated that in kidney cells, overexpression of SGK-1 is protective against ceramide-induced apoptosis and the role of SGK-1 can be potentially explored as a therapeutic target in conditions like diabetes, where ceramide levels are increased.

Apoptosis is a programmed death of cells that has an important role in maintenance of tissue homeostasis by eliminating unneeded cells. Pathways associated with regulation of apoptosis have been demonstrated to contribute in pathophysiology of several diseases like cancer, degenerative diseases, diabetes mellitus (DM) and its associated complications, such as nephropathy.^1^ Among the molecular mechanisms involved in the apoptotic processes, ceramide have a pivotal role.^2, 3^ Sphingolipid ceramide are effector molecules that activate intracellular cascade signalling finally resulting in an anti-proliferative response and apoptosis.^4^ The generation of endogenous ceramide is induced by noxious stimuli such as UVA^5^ and proinflammatory cytokines (e.g., TNF-*α*),^6^ which promote the release of ceramide from sphingomyelin and/or by the ‘*de novo*' synthesis from palmitate.^7^

An enhancement of cellular ceramide leads to reduced activation of insulin signalling and this alteration is characteristic of type 2 diabetic (T2D) patients.^8, 9, 10^ Ceramide induces apoptosis by multiple mechanisms, such as inhibition of AKT-1 and activation of extrinsic and intrinsic apoptotic pathways.^11^ These pathways converge on the cleavage of caspase-3 and result in DNA fragmentation, degradation of cytoskeleton and nuclear proteins, and formation of apoptotic bodies including DNA fragments (‘DNA ladder').^12^ Caspases, a family of cysteine proteases, are expressed in cells as inactive procaspase precursors.^13^ Initiator caspases, such as 8 and 9, are activated by an autocatalytic process induced by the interaction of an initiator caspase, via its pro-domain, with its adaptor protein. Then, initiator caspases cleave the precursor forms of effectors caspases, like caspase-3, which execute apoptosis by cleaving cellular proteins.^14^ Caspases 3 and 7 cleave PARP-1 into two fragments, ~p89 and ~p24, separating DNA binding from the catalytic domain inducing PARP-1 inactive.^15^

Serum and glucocorticoid-inducible kinase-1 (SGK-1) is a 49-kDa AGC family serine threonine protein kinase, displaying similarity (54% homology) throughout its catalytic domains with PKB/AKT-1, however lacking of pleckstrin homology domain (PH), presents in AKT-1.^16^ SGK-1 stimulates a variety of ion channels, transporters and transcription factors, which can modulate kidney function, blood pressure and insulin action, suggesting its role in the pathophysiology of DM and its complications like diabetic kidney disease.^17, 18, 19^ Moreover, SGK-1 has been directly involved in the regulation of glucose uptake.^20^ SGK-1 has been also implicated in the activation of cellular survival pathways since several evidence outline how SGK-1 overexpression and activation inhibit cell apoptosis in mammalian cells, becoming considered a pro-survival kinase.^21^ Furthermore, we showed as SGK-1 also modulates endothelial function by decreasing apoptosis and increasing insulin mediated nitric oxide (NO) production in basal conditions and after oxidative stress damage in endothelial cells.^19^

Therefore, in the present *in vitro* study, we sought to investigate the possible protective role of SGK-1 in the kidney cells against apoptotic damage induced by ceramide.

## Results

### SGK-1 activity is higher in SGK-1wt cells

**Figure 1:**
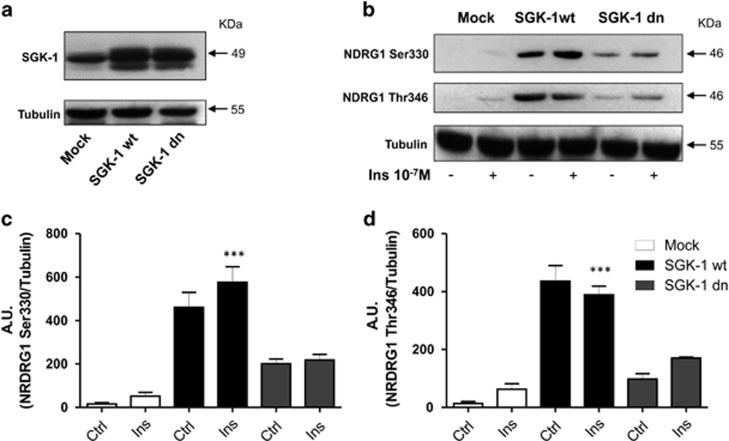
Activity of SGK-1 in HEK-293 cells. Expression of SGK-1 protein is evaluated in HEK-293 cells stable transfected with pcDNA3 (Mock), SGK-1wt and SGK-1dn constructs. 100 *μ*g of total extract protein was analysed by western blot using anti-SGK-1 antibody. Tubulin was used as a loading control (**a**). Activity of SGK-1 in HEK-293 cells stable transfected with pcDNA3 (Mock), SGK-1wt and SGK-1dn constructs was detected through NDRG1 phosphorylation levels in Thr346 and Ser330 in the presence and absence of Insulin 10^−7^ M for 30 min using western blot analysis. Tubulin was used as a loading control. (**b**) A quantification of three independent experiments by scanning densitometry is shown in (**c** and **d**), ***P<0.001 Ins (SGK-1 wt) *versus* Ctrl and Ins (Mock), and Ctrl and Ins (SGK-1 dn); *N*=3. Results are expressed as means±S.D.

Human embryonic kidney (HEK) 293 cells transfected with SGK-1wt and SGK-1dn (dominant negative) constructs were utilized for this study (Supplementary Figure S1). Expression of SGK-1 protein was evaluated in HEK-293 cells transfected with pcDNA3 (Mock), SGK-1wt and SGK-1dn constructs (Figure 1a). We aimed to measure the SGK-1 activity in cell constructs to ensure that transfected cells were biological active. To measure SGK-1 activity, we evaluated the phosphorylation of NDRG1 (N-myc downstream regulated gene 1), a well-established substrate of SGK-1,^22^ in cells transfected with pcDNA3 (Mock), SGK-1wt and SGK-1dn in the presence or absence of insulin for 30 min. Since SGK-1 phosphorylates NDRG1 in Thr346, Thr356, Thr366 and Ser330, we evaluated Thr346 (antibody that cross-reacts with Thr356 and Thr366) and Ser330 phosphorylation site. We found that the phosphorylation of NDRG1 was significantly higher in SGK-1wt cells in the presence and/or absence of insulin compared with SGK-1dn (*P*<0.001) and almost absent in Mock (Figures 1b and d) cells. These results suggest that SGK-1 overexpressed in our wt group is biologically active and present higher activity of this kinase compared with dn cells.

### SGK-1 wt protects against apoptosis induced by C_2_-ceramide

**Figure 2:**
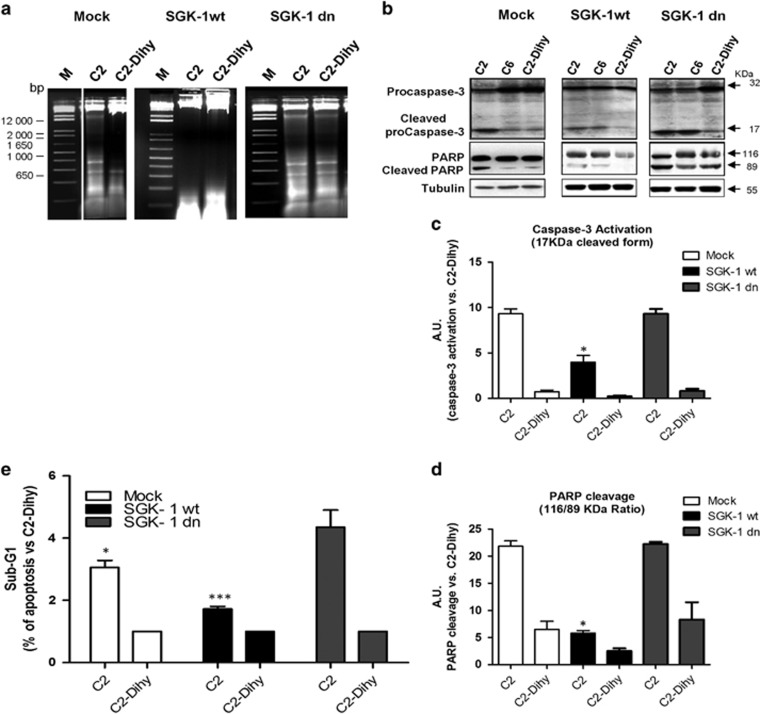
SGK-1wt overexpression protects by ceramide-induced apoptosis. HEK-293 cells stable transfected with pcDNA3 (Mock), SGK-1wt and SGK-1dn constructs were stimulated with 50 *μ*M C_2_-ceramide and C_2_ dihydroceramide (C_2_-Dihy) used as a negative control. Apoptosis was induced with C_2_-ceramide for 48 h and apoptosis levels were detected using DNA fragmentation analysis. The DNA was extracted and analysed on 1.5% Agarose gel (M: marker 1Kb plus) (**a**). The apoptotic effect of C_2_-ceramide *versus* C_2_-Dihy was measured in HEK-293 cells transfected with Mock, SGK-1wt and SGK-1dn by caspase-3 activation and by PARP-1 cleavage (**b–d**). HEK-293 cells stable transfected with Mock, SGK-1wt and SGK-1dn were stimulated with 50 *μ*M C_2_-ceramide, C_6_-ceramide and C_2_-Dihy. After 48 h of treatment with ceramide, the cells were lysed and 100 *μ*g of total proteins was separated on SDS-PAGE and immunoblotted with specific antibodies. Active caspase-3 was identified with antibodies specific for the 17 KDa cleaved fragment while procaspase-3 was identified as 32 KDa protein band. Active, full-length PARP-1 was identified with antibodies specific for the 116-KDa fragment, while cleaved PARP-1 was identified as 89 KDa protein band. Tubulin was used as a loading control. The blots shown are representative of the three independent experiments (**b**). A quantification of three independent experiments by scanning densitometry is shown in (**c** and **d**), *P<0.05 C2 (SGK-1wt) *versus* C2 (Mock and SGK-1 dn); *N*=3. The effect of C_2_-ceramide in HEK-293 cells stable transfected with Mock, SGK-1wt and SGK-1dn was compared by FACS analysis. A representative histogram of apoptosis *versus* C_2_-Dihy is shown in (**e**), ***P<0.001 C2 (SGK-1wt) *versus* C2 (Mock and SGK-1dn), *P<0.05 C2 (Mock) *versus* C2 (SGK-1dn); *N*=3. Results are expressed as means±S.D.

HEK-293 cells were treated with a short chain of synthetic Ceramide (C_2_-ceramide) and the possible noxious effects of C_2_-ceramide were compared with C_2_-dihydroceramide (C_2_-Dihy, inactive analogue) treatments. Cells treated with dimethyl sulphoxide (DMSO, vehicle) and/or C_2_-Dihy maintained normal morphology, whereas cells treated with C_2_-ceramide were rounded, shrunken and partially detached from flask support (Supplementary Figure S2), suggesting as these cells are more susceptible to apoptosis. The intra-nucleosomal DNA fragmentation was measured with DNA-ladder analysis showing higher DNA fragmentation in Mock and SGK-1 dn cells after treatment with C_2_-ceramide compared with SGK-1wt cells group (Figure 2a).

In our model, C_2_-ceramide but not C_2_-Dihy induced caspase-3 activation (revealed by the presence of 17 KDa protein band as active cleaved form) and PARP-1 cleavage (revealed by presence of 89 KDa protein band) (Figure 2b). To further confirm our result, we tested C_6_-ceramide as analogue to C_2_-ceramide. For its conformation, C_6_-ceramide has lower solubility; indeed its effects on caspase-3 activation were reduced compared with C_2_-ceramide treatment in Mock and SGK-1 wt cells, but not in SGK-1 dn cells (Figure 2b). The activation of Caspase-3 and subsequent PARP-1 cleavage were reduced in SGK-1wt cells stimulated with C_2_-ceramide compared with Mock and SGK-1dn cells (*P*<0.05) (Figures 2b and d), suggesting that ceramide-mediated apoptosis is a SGK-1-dependent process. FACS analysis confirmed that SGK-1wt cells have a significant reduction in apoptotic levels compared to cell transfected with SGK-1dn, and empty vector constructs of 2.5- and 2-fold (*P*<0.001), respectively (Figure 2e). Plots of flow-cytometry experiments are reported in Supplementary Figure S3.

These results confirm the protective role of SGK-1 in response to C_2_-ceramide-induced apoptosis.

### C_2_-ceramide increases SGK-1 and decreases AKT-1 activation

SGK-1 protein levels and activation were measured in response to C_2_-ceramide, C_6_-ceramide and C_2_-Dihy. In these experiments, AKT-1 expression and activation levels were also evaluated, since SGK-1 and AKT-1 have similar protective effects after induction of apoptosis.^23^

**Figure 3:**
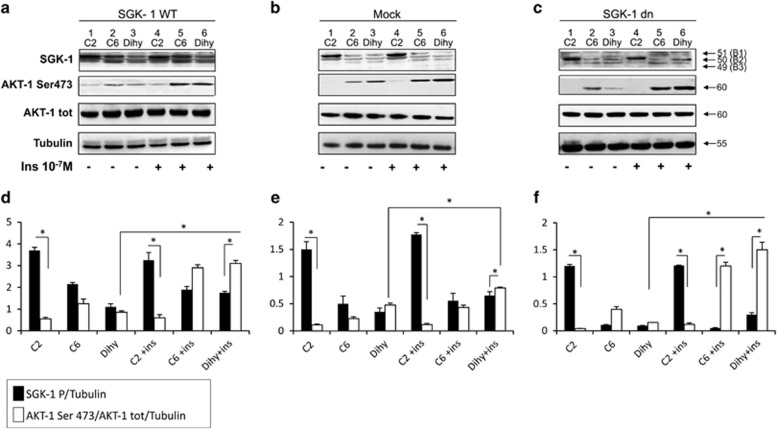
C_2_-ceramide but not C_2_-dihydroceramide activates SGK-1 protein and inhibits AKT-1 phosphorylation and expression. HEK-293 SGK-1wt (**a**), Mock (**b**) and SGK-1dn (**c**) transfected cells were treated with 50 *μ*M C_2_-ceramide (lanes 1 and 4), C6 (lanes 2 and 5) and C_2_-Dihy (lanes 3 and 6), in the presence or absence of insulin 10^−7^M for 48 h. Proteins were analysed by western blot using anti-SGK-1, anti-AKT-1 phospho-Ser-473, anti-AKT-1 total and anti-Tubulin to detect proteins expression and phosphorylation (**a**–**c**). Tubulin was used as a loading control. Western blot analysis and representative densitometry of at least three independent experiments (**d**–**f**), *P<0.05; *N*=3. Results are expressed as means±S.D.

Transfected cells were incubated with C_2_-ceramide, C_6_-ceramide or C_2_-Dihy (negative control), with or without insulin treatment for 30 min to detect SGK-1 and AKT-1 activation. Different SGK-1 protein bands (B1:51, B2:50, B3:49 KDa) were identified as already reported in the literature.^24^ The hyperphosphorylated form of SGK-1 can be considered the slower migrating protein band (B1), while the faster migrating species represent the hypophosphorylated proteins (B2 and B3).^24^ As shown in SGK-1 wt cells (Figure 3), treatment with C_2_-ceramide and C_6_-ceramide increased SGK-1 phosphorylation, while C_2_-ceramide decreased AKT-1 phosphorylation (Ser473) compared to treatment with C_2_-Dihy (*P*<0.05). Furthermore, in the presence of C_2_-ceramide, SGK-1 and AKT-1 phosphorylation was independent of insulin treatment (Figures 3a and d). These results were confirmed in Mock cells even if the protein signal of SGK-1 was lower since SGK-1 in these cells is less expressed (Figures 3b and e). In the SGK-1dn cells, C_2_-ceramide induced synthesis of SGK-1 protein band (B2) with not increased levels of SGK-1 phosphorylation (B1) (Figures 3c and f). The effect of C_2_-ceramide in AKT-1 was even more evident by almost abolishing the protein signal either in the presence or in the absence of insulin treatment (Figure 3). Present results suggest different roles of SGK-1 and AKT-1 in response to C_2_-ceramide independently of insulin treatment. Therefore, we may conclude that the protective action in response to ceramide is due to SGK-1 activation.

### Increased SGK-1 activity and expression is mediated by PKA, p38 and PI3K signalling pathways

**Figure 4:**
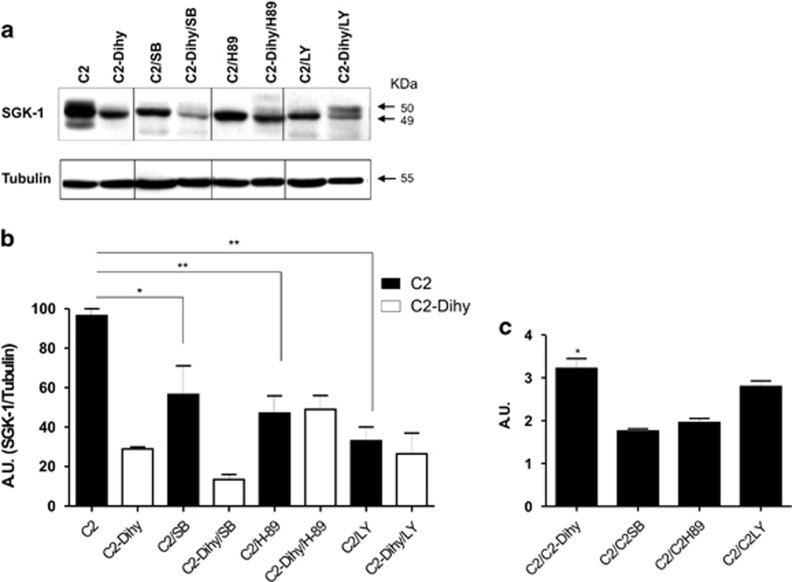
C_2_-ceramide-mediated SGK-1 expression is activated via p38/MAPK, cAMP/PKA and PI3K pathways. HEK-293 cells stably transfected with SGK-1wt were treated with C_2_-ceramide (50 *μ*M), C_2_-Dihy (50 *μ*M), SB202190 (10 *μ*M) (p38 inhibitor), H-89 (10 *μ*M) (PKA inhibitor) and LY294002 (30 *μ*M) (PI3K inhibitor). After 48 h, SGK-1 expression was evaluated with antibodies specific for SGK-1. The protein levels of SGK-1 were compared with Tubulin levels. Results were confirmed in at least three separate experiments. Blots shown are representative of three independent experiments. Tubulin was used as a loading control (**a**). A quantification of three independent experiments by scanning densitometry is shown in (**b**) (*P<0.05, **P<0.01; *N*=3). The Ratio between C_2_-ceramide (C2) and C2-Dihy or C_2_-ceramide with inhibitors (C2/C2SB, C2/C2H89, C2/C2LY) was evaluated (**c**), *P<0.05, C2/C2-Dihy *versus* C2/C2SB, C2/C2H89; *N*=3. Results are expressed as means±S.D.

To further investigate the SGK-1 protective role against apoptosis, we analysed specific pathways regulating SGK-1 expression and activity, which include p38/MAPK, cAMP/PKA and PI3K,^24, 25^ using selective inhibitors SB202190, H-89 and LY294002, respectively.^24, 25, 26, 27^ SGK-1wt cells were treated with inhibitors for 30 min and then cells were stimulated with C_2_-ceramide or C_2_-Dihy (Supplementary Figure S1). In the same experimental conditions, insulin effects were tested to avoid possible bias, and no additional or different modulations among treated groups have been found (data not shown). SGK-1 expression was reduced in the presence of SB202190 (*P*<0.05), H89 (*P*<0.01) and LY294002 (*P*<0.01) after C_2_-ceramide stimulation (Figures 4a and b). Further, confirming the role of C_2_-ceramide, the treatment with C_2_-Dihy, with or without the three different inhibitors, had no effect on the SGK-1 expression (Figures 4a and b). We also calculated (ratio C_2_/C_2_-Dihy, C_2_/C_2_Inhibitors) the decreased expression of SGK-1 in response to C_2_-ceramide in the presence of selective inhibitors, with the aim to understand which is the cell pathway more involved after C_2_-ceramide treatments (Figure 4c). The higher difference in SGK-1 expression was found following treatment with C_2_-ceramide and LY inhibitor, suggesting a principal role of PI3K pathway in modulation of SGK-1 levels.

### SGK-1 protects against apoptosis induced by TNF-*α*

**Figure 5:**
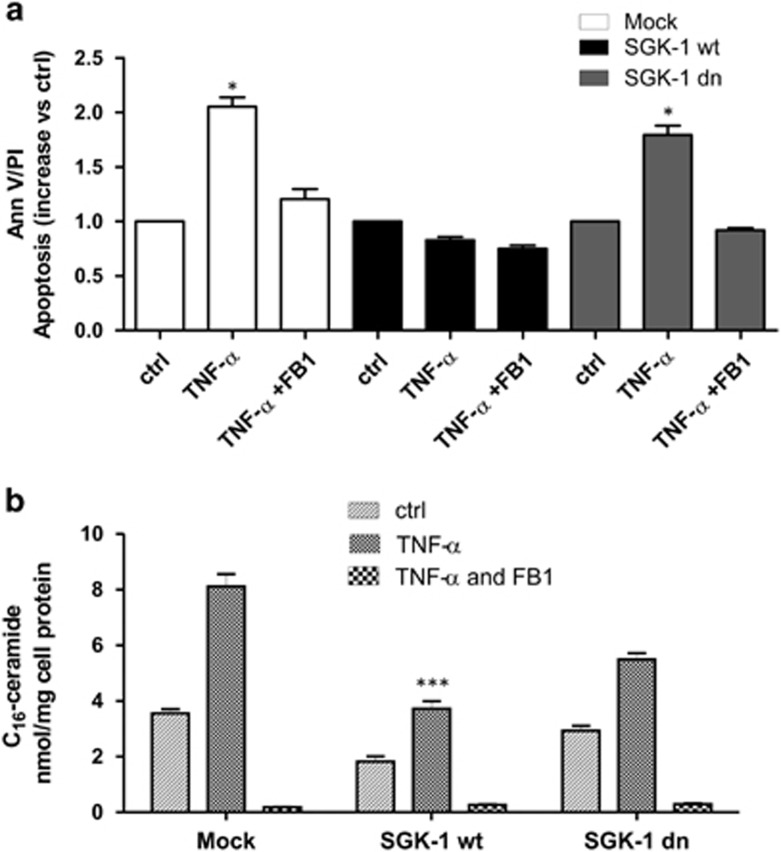
Effect of TNF-*α* on protection mediated by SGK-1. HEK-293 cells were transfected with Mock, SGK-1wt and SGK-1dn constructs. The cells were treated with TNF-*α* (100 ng/ml) for 72h in the presence or absence of 100 *μ*M FB1 (Fumonisin-B1) to inhibit ceramide production. FACS analysis as representative histogram of apoptosis *versus* ctrl (**a**), *P<0.05 TNF-*α* (Mock and SGK-1 dn) *versus* TNF-*α*+FB1 (Mock and SGK-1 dn), *P<0.05 TNF-*α* (Mock and SGK-1 dn) *versus* TNF-*α* (SGK-1 wt); *N*=3. Sphingolipid long-chain bases (C_16_-ceramide) were measured by gas chromatography/mass spectrometry (GC/MS) (**b**), ***P<0.0001 SGK-1wt (TNF-*α*) *versus* Mock (TNF-*α*) and SGK-1dn (TNF-*α*); *N*=3. Results are expressed as means±S.D.

Since TNF-*α* stimulus increases the level of cellular ceramide^28, 29^ transfected cells were treated with TNF-*α* for 72 h (Supplementary Figure S1). SGK-1wt have a significant lower percentage of apoptosis compared with Mock and SGK-1dn groups after TNF-*α* stimulation (~2-fold, *P*<0.05) (Figure 5a). The activation of *‘de novo'* ceramide synthesis leads to ceramide-induced apoptosis. Therefore, we also investigated whether inhibition of this process protects cells from ceramide-induced apoptosis. HEK-293 were treated with or without Fumonisin B1 (FB1), an inhibitor of ceramide synthase, and then stimulated with TNF-*α* (Supplementary Figure S1). In SGK-1dn and Mock cells, the treatment with FB1 30 min before TNF-*α* exposure induced a significant reduction of apoptosis, suggesting that it was mediated, at least in part, by *‘de novo'* ceramide synthesis (Figure 5a). FB1 alone did not induce any significant effect in HEK-293 (data not shown).

To confirm our hypothesis, we measured levels of endogenous ceramide after TNF-*α* treatment. Elevated levels of ceramide were observed in Mock and SGK-1dn cells, while in SGK-1wt cells a significant reduction of ceramide with respect to Mock and SGKdn was found (*P*<0.0001). TNF-*α-*treated cells were characterized by production of C_16_-ceramide. FB1 treatment induced a significant TNF-*α*-dependent reduction of C_16_-ceramide in all transfected groups (Figure 5b). Apoptotic process was confirmed measuring PARP-1 cleaved levels (Supplementary Figure S4). Indeed, SGK-1wt cells were more resistant to apoptosis after TNF-*α* treatments *versus* Mock (*P*<0.05), and SGK-1dn cells (*P*<0.05).

### Selective SGK-1 inhibitor blunts apoptotic protection

**Figure 6:**
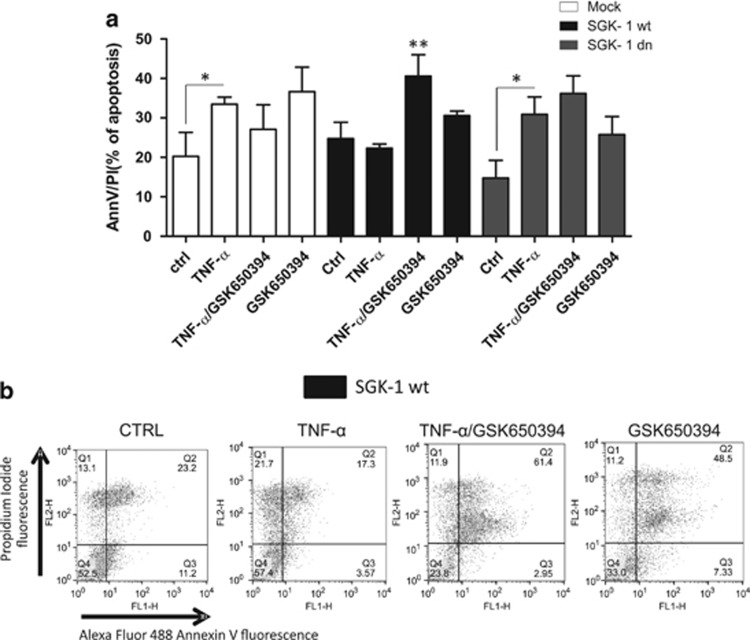
Effect of SGK-1 inhibition on protection against apoptosis. HEK-293 cells were transfected with Mock, SGK-1wt and SGK-1dn constructs and stimulated with SGK-1 inhibitor, GSK650394 (103 nM), 60 min before TNF-*α* treatment (100 ng/ml) for 72 h (**a**). Apoptotic cells were determined by annexin V-FITC/PI method. These results were obtained from at least three different experiments (**a**), **P<0.01 TNF-*α*/GSK650394 (SGK-1 wt) *versus* TNF-*α* (SGK-1 wt), *P<0.05 TNF-*α* (Mock, SGK-1 dn) *versus* ctrl (Mock, SGK-1 dn); *N*=3. Results are expressed as means±S.D. The plots of (**b**) show a typical flow-cytometry experiment in SGK-1 wt constructs (**b**)

To test whether SGK-1 is pivotal in the protection against apoptosis induced by ceramide, we treated the three groups with a selective inhibitor of SGK-1 activity (GSK650394) 60 min before TNF-*α* stimulation.^30^ After 72 h, cells were analysed by FACS (Supplementary Figure S1). SGK-1wt cells after TNF-*α* stimulation showed reduced levels of apoptosis compared with SGK-1dn (*P*<0.05) or Mock cells (*P*<0.05) (Figures 6a and b). After treatment with GSK650394, no significant differences in apoptotic levels were present either in Mock or in SGK-1dn cells, while the inhibitor significantly blunted the protection against apoptosis in the SGK-1wt (*P*<0.01) cells, suggesting a pivotal role of this kinase in protection against ceramide insult.

### TNF-*α* induces apoptosis through caspase-3-dependent pathways

**Figure 7:**
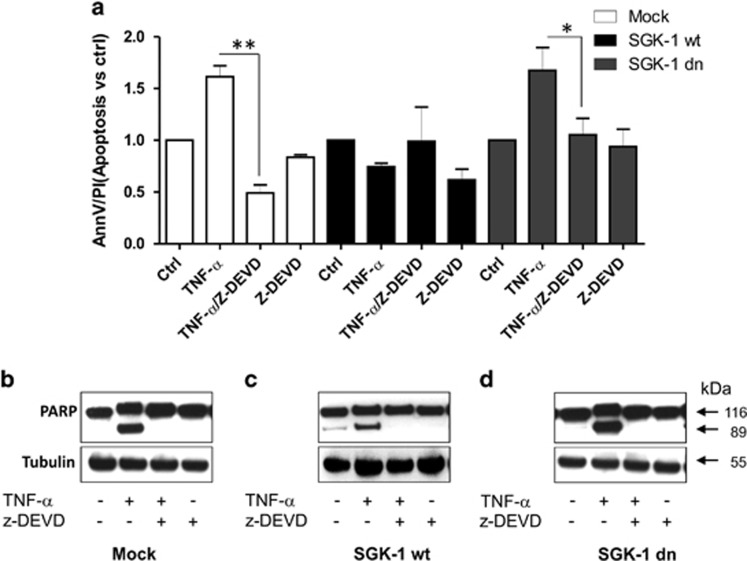
Apoptosis induced by TNF-*α* is mediated via caspase-3 activation. HEK-293 cells were transfected with Mock, SGK-1wt and SGK-1dn constructs and stimulated with caspase-3 inhibitor, Z-DEVD-FMK, (20 *μ*M), 60 min before TNF-*α* treatment (100 ng/ml) for 72 h. Apoptotic cells were determined by annexin V-FITC/PI method (**a**). After 72 h of treatment with TNF-*α*, the cells were immunoblotted with specific antibodies for full-length (116 KDa) and cleaved PARP-1 (89 KDa). Tubulin was used as a loading control. Blots shown are representative of three independent experiments (**b–d**). These results were obtained from at least three different experiments, **P<0.01TNF-*α versus* TNF-*α*/Z-DEVD (Mock), *P<0.02 TNF-*α versus* TNF-*α*/Z-DEVD (SGK-1 dn); *N*=3. Results are expressed as means±S.D.

We aimed to investigate whether caspase-3 was also involved in apoptosis induced by TNF-*α* stimulus in our experimental condition. We treated transfected cells with selective caspase-3 inhibitor (Z-DEVD-fmk) 60 min before TNF-*α* stimulation. After 72 h, cells were analysed by FACS (Supplementary Figure S1). TNF-*α* induced greater levels of apoptosis in Mock and in SGK-1dn cells compared with control (*P*<0.05), while lower apoptotic stimulus was present in SGK-1wt cells (Figure 7a). Treatment with Z-DEVD-fmk significantly reduced numbers of apoptotic cells either in Mock (*P*<0.01) and in SGK-1dn (*P*<0.02) (Figure 7a). No effect of Z-DEVD-fmk was present in SGK-1wt cells as the apoptotic stimulus induced by TNF-*α* was counteracted by SGK-1 (Figure 7a). These data confirmed the role of caspase pathways activation in apoptosis mediated by ceramide, and the protective role of SGK-1 in response to these stimuli, which induce ceramide production.

As previously introduced, PARP-1 is also a caspase-3's substrate in the apoptotic pathway. In our model, we demonstrated higher levels of PARP-1 cleavage after treatment with TNF-*α* in Mock and SGK-1dn cells, which was abolished with caspase-3 inhibitor. Representative WB images are reported in Figures 7b and d. This effect was significantly (*P*<0.05) reduced in the SGK-1wt group.

Since the activation of caspase-3 depends on activation of caspases 8 and 9 through 2 distinct pathways,^31^ we investigated their role in our experimental model of apoptosis. Both caspases 8 and 9, when activated by TNF-*α* and when blocked by selective inhibitors (Z-IETD and Z-LEHD, respectively), had similar effect in the different transfected cellular groups, with no significant variation among them (Supplementary Figure S5). Therefore, our data confirmed that both intrinsic and extrinsic pathways of apoptosis participating in the activation of caspase-3, and then in the related apoptosis activation.

## Discussion

**Figure 8:**
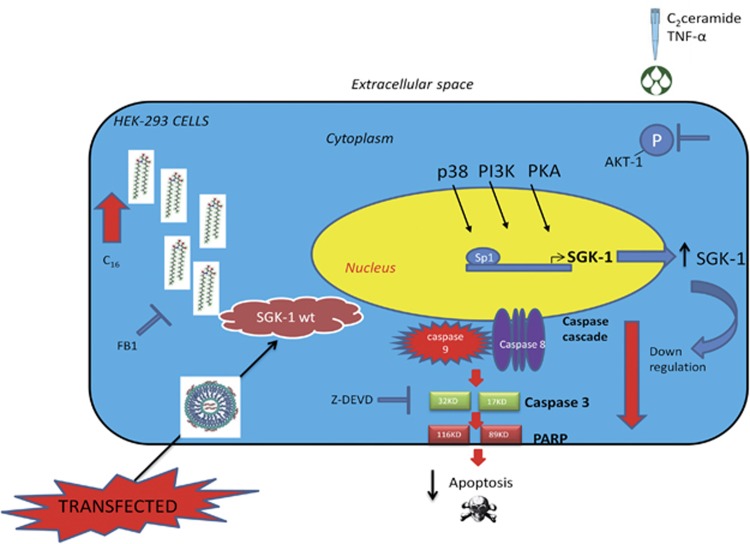
Schematic diagram of pathways activated by ceramide and TNF-*α* inducing SGK-1 activation and protection against apoptosis. C_16_: C_16_-ceramide, FB1: fumonisin B1, HEK-293: human embryonic kidney, PI3K: phosphatidylinositide 3-kinases, PKA: protein kinase A, p38: P38 mitogen-activated protein kinases, PARP: poly (ADP-ribose) polymerase, SGK-1 wt: serum and glucocorticoid inducible protein kinase wild type, Z-DEVD: caspase-3 inhibitor

In the present study, we suggested a protective role of SGK-1 against apoptosis induced by ceramide and TNF-*α* in kidney cells overexpressing SGK-1. The anti-apoptotic action of active SGK-1, tested by measuring NDRG1 phosphorylation levels, was mediated by reduction of caspases activation (caspases 3, 8 and 9) and inhibition of intrinsic and extrinsic apoptotic pathways in response to TNF-*α* with reduced levels of PARP-1 cleavage. Moreover, a different response of SGK-1 and AKT-1 following apoptotic insult has been described in this study, identifying biological differences between these two kinases. In Figure 8 is reported the representation of the investigated pathways. The described results propose a novel protective role of SGK-1 against apoptosis induced by ceramide and TNF-*α*, increasing our knowledge in this field, opening new perspectives to counteract cellular and tissue injuries, which are typical of several diseases like diabetes and its complications.^10^

SGK-1 has been shown to regulate different cellular pathways in response to harmful stimuli, including phosphorylation of Foxo3a with its consequent translocation from nucleus to cytoplasm, finally resulting in a reduction of pro-apoptotic gene transcriptions.^32^ Recently, we demonstrated an increased SGK-1 expression and activity resulted in higher production of NO, inhibition of reactive oxygen species (ROS) synthesis, and lower apoptosis in endothelial cell after hyperglycaemia.^19^ Moreover, in cells overexpressing SGK-1 we found an enhancement in GLUT-1 membrane translocation and Na^+^-K^+^ ATPase activity.^19^ These results suggest as in endothelial cells higher SGK-1 activity reduced oxidative stress, improved cell survival and restored insulin-mediated NO production.^19^

Nephropathy represents one of the major complications in diabetes, characterized by apoptosis of renal mesangial cells.^33, 34^ Increased expression of SPT (serine palmitoyl transferase), an enzyme involved in ceramide synthesis, was seen in renal tubular epithelial and microvascular endothelial cells, which are the main sites of apoptosis observed in diabetic patients.^35^ When ceramide generation was inhibited using SPT inhibitors and ceramide synthase inhibitors (FB1), a reduction in tubular epithelial cell death was observed.^11, 36, 37^ In this study, we confirmed the apoptotic action of ceramide in response to TNF-*α*, by inhibiting ceramide synthase. Moreover, we demonstrated a pivotal role of SGK-1 in this process that, when inhibited by using its selective inhibitor, resulted in increased apoptotic levels. Interestingly, previous studies conducted in HEK-293 reported a protective role of SGK-1 against apoptosis induced by hypoxic renal injury,^38^ suggesting that one of the possible mechanisms linked SGK-1 protection *versus* ischaemia is modulation of erythropoietin production.^39^ We used C_2_-ceramide and TNF-*α* as a model of injury more related to metabolic disorders. It is known that both stimuli induce apoptosis by activating the cascade of caspases and their substrates.^40, 41, 42, 43, 44^ We demonstrated that overexpression and activation of SGK-1 reduced the cleavage of caspase-3 after C_2_-ceramide stimulus and inactivation of caspase-3 substrate (PARP-1), protecting against apoptosis. We showed, for the first time, a direct relationship between SGK-1 and caspase's cascade. Particularly, by inhibiting specific pathways, we reported a direct effect of caspase-3 in SGK-1-mediated cellular protection. We also established, after TNF-*α* treatment, using selective caspase inhibitors, that both initiator caspases 8 and 9 have a principal role in activation of apoptosis in response to TNF-*α*. It is possible to speculate that intrinsic and extrinsic pathways are triggered, as caspase-9 is activated because of mitochondrial damage and cytochrome C release and caspase-8 is activated by death receptors to initiate the extrinsic pathway of apoptosis.^14^ In agreement with our data, it was reported that an increase in SGK-1 was protective in neurons against traumatic brain injury by GSK3*β*/*β*-catenin pathway, which ultimately leaded to inhibition of caspase-3 activation.^45^

Among mechanisms mediated by increased ceramide production, the inhibition of AKT-1, a powerful promoter of cell survival, can partly explain the pro-apoptotic actions of ceramide.^11^ Furthermore, AKT-1 is a principal substrate in the pathways linking insulin to expression, action and subcellular distribution of nutrient transporters.^46^ Indeed, higher production of ceramide can increase insulin resistance through different mechanisms and modulate the pathogenesis of diabetes.^47, 48^ Since now, the role of SGK-1 in the regulation of these actions was unclear, although its high homology with AKT-1. Indeed, in response to ceramide we found an increased activation and expression of SGK-1, but differently a reduced activation and expression of AKT-1, demonstrating a different role of these kinases. C_2_-ceramide has been already shown to decrease activation of AKT-1 increasing apoptosis in neurons.^49^ In fact, several findings suggest the protective role of AKT-1 against apoptosis and constitutive active AKT-1 can inhibit caspases 9 and 3 by post-translational modifications.^50^ We may speculate that differences in the protective response between SGK-1 and AKT-1 could be tissue specific, mediated and dependent on the characteristics of the noxious stimulus, in a compensatory mechanism. In agreement with this hypothesis, the different action of ceramide on SGK-1 and AKT-1 may be, at least in part, dependent on the absence of pleckstrin homology (PH) domains in SGK-1, which is pivotal for the interaction of PDK-1 and AKT-1 with plasma membrane phosphoinositides, modulating its activation.^21, 47^ Further studies are needed to investigate these assumptions.

Differential expression of SGK-1 at the cellular and subcellular levels contributes to signalling specificity.^51^ An excessive SGK-1 expression and activity has been shown to participate in different manner in the pathophysiology of several disorders.^51^ Indeed, the role of SGK-1 in inducing protection is pretty controversial and debated. In kidney, SGK-1 assumes distinct roles in regulating Na^+^ and K^+^ transport through the activation of epithelial Na^+^ channel (ENaC) opening, Na^+^-K^+^-ATPase and K^+^ channels including ROMK, mainly in principal cells of distal tubules, which are sensitive to aldosterone. SGK-1 levels can augment in response to aldosterone increasing kidney Na^+^ reabsorption and K^+^ excretion and therefore affecting blood pressure.^52^ In agreement with these studies, we showed SGK-1-mediated Na^+^ regulation in HUVEC.^19^ Currently, to clarify the pathophysiological role of SGK-1, pharmacological inhibitors of SGK-1 could be pivotal to reveal utility of this kinase as a therapeutic target.

Several are the pathways regulating the activity of SGK-1.^53^ Among those, the most important are cAMP/PKA, p38MAPK and PI3K pathways, which when activated increase SGK-1 cellular levels,^53, 54^ as further demonstrated by the results of the present study. Interesting, SB202190 by blocking p38MAPK can regulate SGK-1 expression at transcriptional level, probably by binding Sp1 transcription factor to SGK-1 promoter (Figure 8).^25, 26^ Among all tested inhibitors, LY294002 had the greater effect on the expression of SGK-1, suggesting as the PI3K pathway deserves further investigation. We also used SGK-1 selective inhibitor (GSK650394) to test the cellular reaction in response to apoptotic stimuli after direct inhibition of SGK-1 kinase activity. The inhibition of SGK-1 resulted in higher levels of cellular death. Our data are in line with previous reports showing higher insulin resistance and cellular damage in cortical collecting duct cells treated with GSK650394.^55^

These results and our previous study^19^ suggest a protective role of SGK-1 in kidney and endothelial cells by different cellular mechanisms. The translation of our results in human by testing the effect of specific SGK-1 modulators could be of great interest since genetic variants of SGK-1 have been already found associated with risk of hypertension^56^ and T2D.^57^ Moreover, a specific SGK-1 polymorphism (rs9493857) has been suggested to be one of the ancestral allele associated with different responses to stress in humans.^58^ Therefore, different SGK-1 genetic variants may code for different type of kinases with higher or lower predisposition to be ubiquitinated and then inactivated, based on their amino acidic sequences, and therefore with different abilities to respond to noxious stimuli.

Strengths of the present study include well-established experimental models by the use of specific cellular constructs, which allow investigating different levels and activities of the kinase. We also employed selective inhibitors for SGK-1 and for the cellular pathways involved in its expression and regulation. Moreover, to our knowledge, we are the first demonstrating the SGK-1 protection against apoptosis mediated by both hexogen and endogen ceramide. We have to acknowledge some limitations for this study. Although overexpressed the kinase still undergo to the ubiquitination process and therefore we are not able to fully control the exact levels of SGK-1 after stimulation. However, this mechanism, even if recognized as a limitation, mimics the physiological cellular process. Other limitations can be, in general, associated with the use of experimental *in vitro* models that are artificial non-physiological conditions. The employment of SGK-1 knockdown would have been strongly strengthen the conclusion of this paper. However, previous evidence already demonstrated as siSGK-1 leads to enhancement of apoptosis in different conditions.^59, 60^

In conclusion, in the present study, we demonstrated as overexpression of SGK-1 kinase is protective against apoptotic insult induced by ceramide in kidney cells. Since aberrant ceramide accumulation in peripheral tissues contributes to the development of pathological clinical manifestations associated with diseases such as diabetes mellitus, obesity, insulin resistance, atherosclerosis and hypertension,^61^ an important future therapeutic strategy could be to develop inhibitors of ceramide action. Therefore, further studies are needed to better define the therapeutic role of SGK-1 in this field.

## Materials and Methods

### Cell culture

HEK-293 cells were cultured in Dulbecco's Modified Eagle's Medium supplemented with 100 U/ml penicillin/streptomycin (Invitrogen, Milan, Italy) and 10% fetal bovine serum (Invitrogen). Cells were propagated at 37 °C in humidified air containing 5% CO_2_ in 75 cm^2^ culture flasks and subcultured when the cells reached 70–80% confluence (every 2–3 days).

### Plasmid constructs

SGK-1wt (*Eco*RI, *Xba*I) and SGK-1 dominant negative (dn) (Asp222Ala) (BAMHI, *Not*I) were cloned in the expression vector pcDNA3 (Clontech, Saint-Germain-en-Laye, France).

### Plasmid transfection

HEK-293 cells were plated in 100 mm culture vessel and at 50–90% of confluence were transfected with expression plasmids pcDNA3 Myc-SGK-1wt, pcDNA3 Myc-SGK-1dn or pcDNA3 empty vector (Mock) constructs (4 *μ*g/plate) using LipofectAMINE PLUS (Life Technologies, Milan, Italy) following the manufacturer's instructions. For stable expression, the cells were selected with genetycin G418 400 *μ*g/ml. Several weeks of selection were required for stable expression.

### Protein extraction and western blot analysis

HEK-293 cells, incubated with the different experimental conditions, were collected and lysed. Proteins were extracted with lysis buffer pH 7.2 containing EDTA 5 mM, sodium orthovanadate 2 mM, NaF 100 mM, NaPP 5 mM, Tris-HCl pH 7.6, 50 mM, Triton 1%, PMSF 1 mM and complete protease inhibitor 25X (Roche, Basel, Switzerland). Lysate was sonicated for 10 s and centrifuged for 30 min at 13 000 r.p.m./4 °C. The protein concentrations were quantified using Bradford protein assay according to the manufacturer's instructions. For WB analysis, an equal amount of proteins (100 *μ*g) was loaded on 10% SDS-polyacrylamide gel and transferred onto a nitrocellulose membrane (Millipore, Darmstadt, Germany) by electroblotting. After 1 h of blocking with 5% milk, the blots were probed and incubated with the specific antibodies overnight at 4 °C. Then, blots were treated with diluted enzyme-linked secondary antibodies, and visualized by enhanced chemioluminescence (ECL-PE, Amersham, Chalfont Saint Giles, UK) according to the manufacturer's procedure. Primary antibodies used were polyclonal *anti-PARP-1* (89 and 116 KDa), anti-rabbits, 1 : 1000 in WB (Cell Signalling, Danvers, MA, USA), polyclonal *anti-SGK-1* (50 kDa) anti-rabbits, 1 : 1000 in WB (UpState in Millipore), polyclonal *anti-AKT-1*, (60 kDa) anti-rabbits, 1 : 1000 in WB (Cell Signalling), polyclonal *p-AKT-1 (Ser 473)* (60 kDa), anti-rabbits, 1 : 1000 in WB (Cell Signalling), monoclonal *anti-caspase-3 (clone 4-1-1-18)* (32  and 17 KDa) anti-mouse 1 : 1000 in WB (UpState in Millipore), monoclonal *anti-tubulin (clone DM 1 A)* (55 kDa) anti-mouse 1 : 10 000 in WB (Sigma, Milan, Italy), *anti-p-NDRG1 (Thr346 and Ser 330)* (46–48 kDa) anti-rabbits, 1 : 1000 in WB (Cell Signalling).

### Cell treatments

*Insulin* (Sigma): 10^−7^M for 30 min. *C*_*2*_*-Ceramide/C*_*6*_*-ceramide/C*_*2*_*-Dihydroceramide* (BIOMOL in Enzo Life Science, Farmingdale, NY, USA): dissolved in DMSO (vehicle), then diluted into serum-free DMEM at the indicated concentrations and briefly sonicated, concentration used in the experiment is 50 *μ*M, in serum-free medium in 0.1% BSA RIA Grade (Sigma) for 48 h. *TNF-α* (Sigma): 100 ng/ml for 72 h. *FumonisinB1* (Cayman Chemical Company, Ann Arbor, MA, USA): 100 *μ*M, 30 min before the stimulation with TNF-*α*. *LY294002* (Calbiochem, San Diego, CA, USA): 30 *μ*M, 30 min before the stimulation with insulin, C_2_-ceramide or C_2_-Dihydroceramide*. SB202190, H-89* (Calbiochem): 10 *μ*M, 30 min before the stimulation with insulin, C_2_-ceramide or C_2_-Dihydroceramide. *GSK650394* (Tocris, Bristol, UK): 103 nM, 60 min before the TNF-*α* stimulation. *Z-DEVD-fmk* (BD Pharmingen, Milan, Italy): 20 *μ*M, 60 min before the TNF-*α* stimulation.

### DNA ladder analysis

HEK-293 cells were transfected with expression plasmids pcDNA3 Myc-SGK-1wt, pcDNA3 Myc-SGK-1dn and pcDNA3 empty vector constructs, after 24 h were stimulated with C_2_-ceramide or C_2_-dihydroceramide for 48 h. Then, cells were collected and lysed in 10 mM NaCl, 100 mM EDTA, 1% SDS and 200 mM Tris-HCl pH 8.5 buffer. Lysates were treated for 2 h at 60 °C with 100 μg/ml proteinase K. Then, samples were centrifuged at 13 000 r.p.m. for 15 min at 4 °C, supernatants were transferred to a new tube and treated with RNase-DNase free 100 μg/ml at 50 °C for 40 min. The DNA was extracted by phenol/chloroform (1 : 1). DNA was separated through 1.5% agarose gel and stained with ethidium bromide (EtBr, Sigma). Finally, DNA fragmentation pattern was visualized by ultraviolet light source.

### Flow-cytometry analysis for measurement of sub-G1 phase

Apoptotic cell nuclei were detected in HEK-293 cells by flow cytometry. Briefly, cells were harvested, centrifuged at 1000 r.p.m. for 5 min and washed twice with 0.5 ml of PBS. Then, cell lysates were gently resuspended in 0.5 ml propidium iodide (PI) hypotonic solution (50 *μ*g/ml in 0.1% sodium citrate added in 0.1% Triton-X 100) (Sigma) and incubated for 15 min in darkness. The fluorescence emitted from PI–DNA complex was quantified after laser excitation (488 nm) using a FACScalibur (Becton Dickinson, Milan, Italy) and data were analysed by BD-CellQuest software (Milan, Italy). Experiments were carried out on about 10 000 cells and repeated 3–5 times.

### Fluorescence-activated cell Sorter analysis using AnnexinV/PI Staining

A total of 5 × 10^5^ cells were harvested and washed with PBS. To detect apoptotic changes in the position of phosphatidylserine, annexin V binding assay was performed using the Annexin V-FITC/PI apoptosis detection kit (BD Pharmingen) according to the manufacturer's protocol. The procedure consists of the binding of annexin V-FITC to phosphatidylserine in the membrane of cells, which are beginning the apoptotic process, and the binding of PI to the cellular DNA in cells where the cell membranes were been totally compromised. The cells are incubated with annexin V-FITC and PI. After 10 min of incubation at room temperature, cells were analysed by flow cytometry. Annexin V-FITC was detected as a green fluorescence and PI is detected as a red fluorescence. Ten thousand events generally were monitored, and data analysis was performed using BD-Cell Quest software.

### Ceramide and sphingomyelin species analysis in HEK 293 cells

Simultaneous identification of ceramide and sphingomyelin species was performed by gas chromatography/mass spectrometry (GC/MS). Separation of ceramide from sphingomyelin species was obtained by ‘on-injector' thermal cracking of trimethylsilyl (TMS)-derivatized sphingolipids as described by others^62^ with some modifications. Briefly, cultured cells were scraped in cold PBS and sonicated. Cell proteins content was assayed and 5 *μ*g of internal standard (IS) (C_17_ CER, C17:0 S18:1) was added to 250 *μ*g of cell lysate. Lipids were extracted according to Bligh and Dyer^62^ and TMS derivatized by incubating overnight the lipid extract with 25 *μ*l of a BSTFA (1% trimethylchlorosilane, TMCS)-acetonitrile (1 : 1 v/v) mixture. Two microliters of sample were injected on a HP1313 low-polarity column (Agilent Technologies, Santa Clara, CA, USA) in a Agilent HP5890 gas chromatograph under the following conditions: injector temperature: 310 °C; oven: starting at 225 °C for 1 min, then the temperature increased to 325 °C at 5 °C/min and remained stable for 10 min. The m/z ratios were collected by an Agilent HP5973N mass spectrometer in single ion monitoring (SIM) mode. The 311 m/z could be used as the measuring ion since it exhibited the best peak purity as demonstrated by injecting increasing amount of a standard mixture spiked on a cell matrix (data not shown). The 370 and 311 m/z ratios were used for the absolute quantification of the C_16_- ceramide. A calibration curve was built by spiking cell lysate replicates, obtained from a large pool, with 5 *μ*g of IS and increasing amounts of true C_16_-ceramide standard (Avanti Polar Lipids, Alabaster, AL, USA). C_16_-ceramide curve was highly linear (Pearson *R*>0.99) between 0.3 and 5 *μ*g/l of injected ceramide (data not shown). All standards and reagents other than indicated were purchased from Sigma-Aldrich (St Louis, MO, USA).

### Microscope images

Cell images were performed by a microscope Nikon Eclipse TE-2000S (Nikon, Florence, Italy) equipped with a cool-snap camera (see Supplementary Information).

### Statistical analysis

Each experiment was repeated at least three times with reproducible results consistently. Data are expressed as mean±S.D. of combined results from three independent experiments. Statistical analysis was performed using one-way ANOVA test followed by the *post hoc* test of Newman–Keuls or with the unpaired *t*-test. Differences at *P*<0.05 were considered as statistically significant.

## Abbreviations

C2: C_2_-ceramide
C6: C_6_-ceramide
cAMP: cyclic adenosine monophosphate
C2-Dihy: C_2_-dihydroceramide
CTRL: control
DM: diabetes mellitus
DMSO: dimethyl sulphoxide
ENaC: epithelial Na^+^ channel
FB1: Fumonisin B1
GC/MS: gas chromatography/mass spectrometry
GLUT-1: glucose transporter 1
GSK: GSK650394
HEK-293: human embryonic kidney
HUVEC: human umbilical vein endothelial cells
Ins: insulin
IS: internal standard
NDRG1: N-myc downstream regulated gene 1
NO: nitric oxide
p38: P38 mitogen-activated protein kinases
PARP-1: poly (ADP-ribose) polymerase-1
PBS: phosphate buffer saline
PDK-1: phosphoinositide-dependent kinase-1
PH: Pleckstrin homology domain
PI: propidium iodide
PI3K: phosphoinositide-3 kinase
PKA: protein kinase A
PKB: protein kinase B
ROS: reactive oxygen species
SDS-PAGE: sodium dodecyl sulphate-polyacrylamide gel electrophoresis
SGK-1: serum and glucocorticoid inducible protein kinase
SGK-1dn: SGK-1 dominant negative
SGK-1wt: SGK-1 wild type
SIM: single ion monitoring
siRNA: short interfering RNA
SPT: serine palmitoyl transferase
T2D: type 2 diabetes
TMS: trimethylsilyl
TNF-*α*: tumour necrosis factor-alpha
WB: western blot
