# Supplementary material for: SGK-1 protects kidney cells against apoptosis induced by ceramide and TNF-α
Source: Cell Death Dis. 2015 Sep 17;6(9):e1890–. doi: 10.1038/cddis.2015.232 (PMC4650437; doi:10.1038/cddis.2015.232)
Supplement: Supplementary Information [file cddis2015232x1.pdf]

Supplementary Information

The Supplemental Information include:

Figure (S1): Experimental Design.

Figure (S2): The morphology of the cells.

Figure (S3): Plots of typical flow cytometry experiments.

Figure (S4): The effect of TNF-α on PARP-1 cleavage.

Figure (S5): The measurement of apoptosis following stimulation with TNF-α and/or caspases -8 and -9 inhibitors.

Supplementary Figures S1-S5

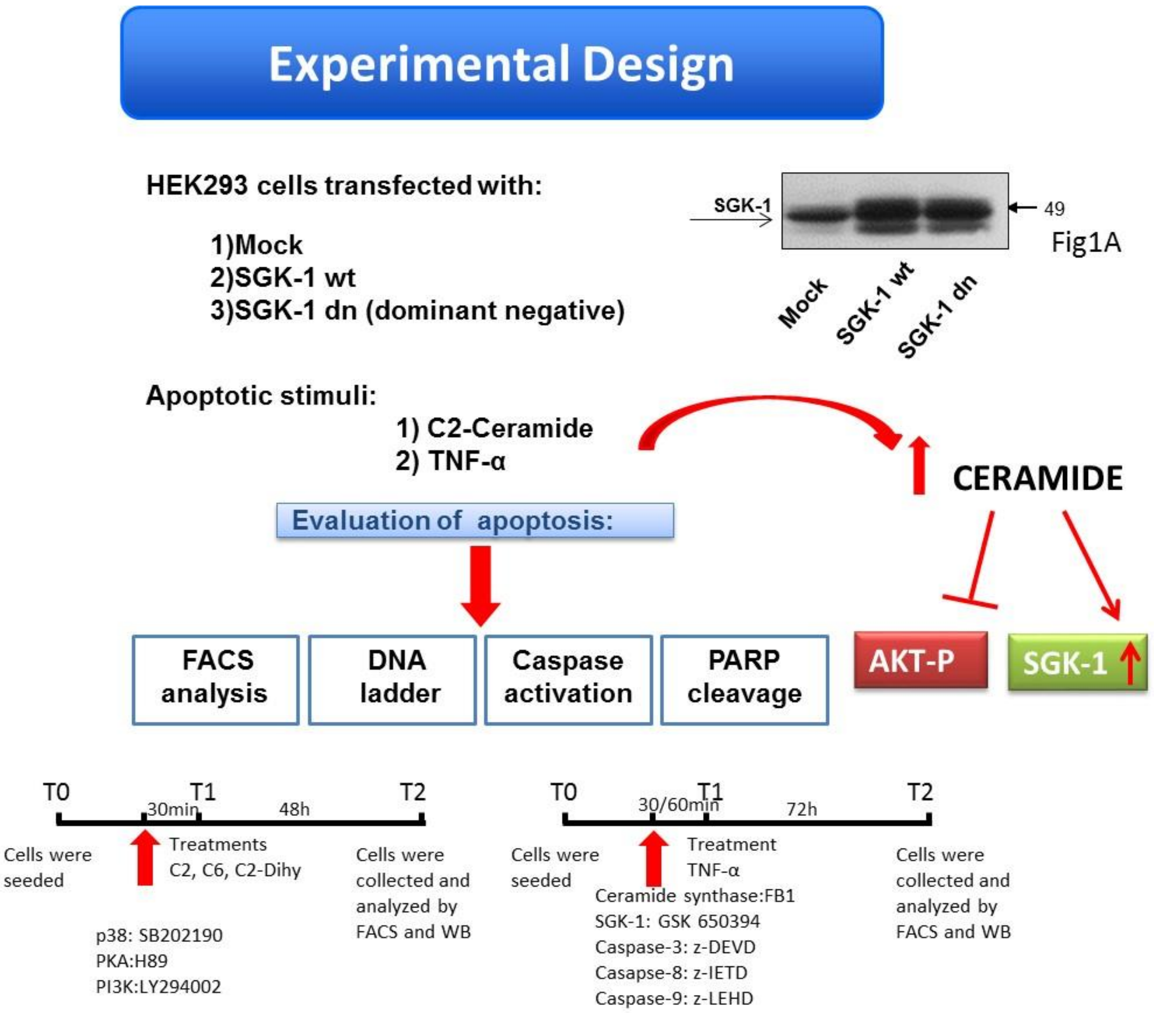

Supplementary Figure S1: Schematic representation of the experimental protocol.

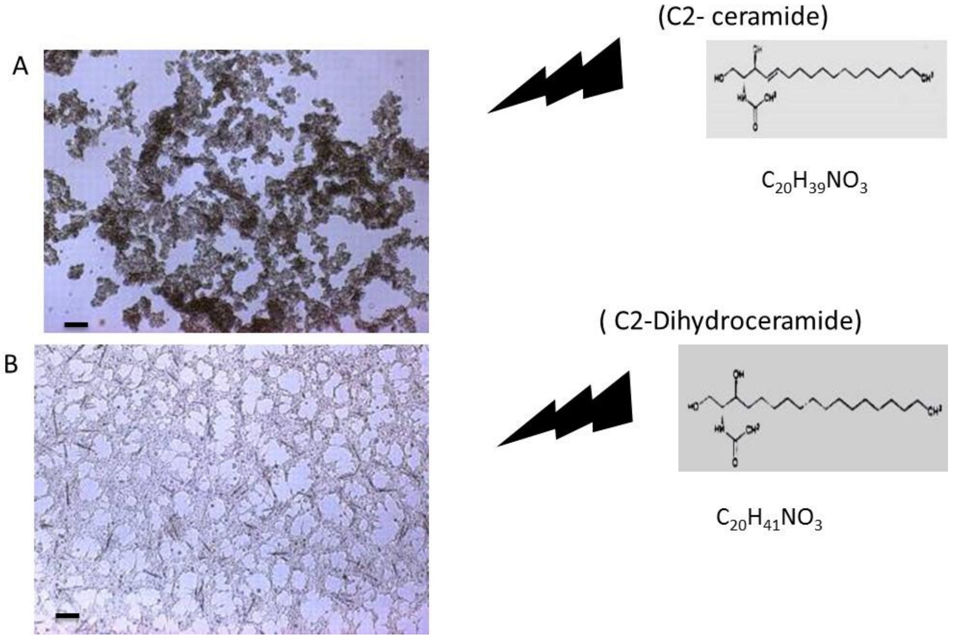

Supplementary Figure S2: Microscope images of cells stimulated with C_2-ceramide or C_2-Dihydroceramide. Space bar = 100 µm (**Panels A, B**). Cell images were performed by a microscope Nikon Eclipse TE-2000S equipped with a cool-snap camera.

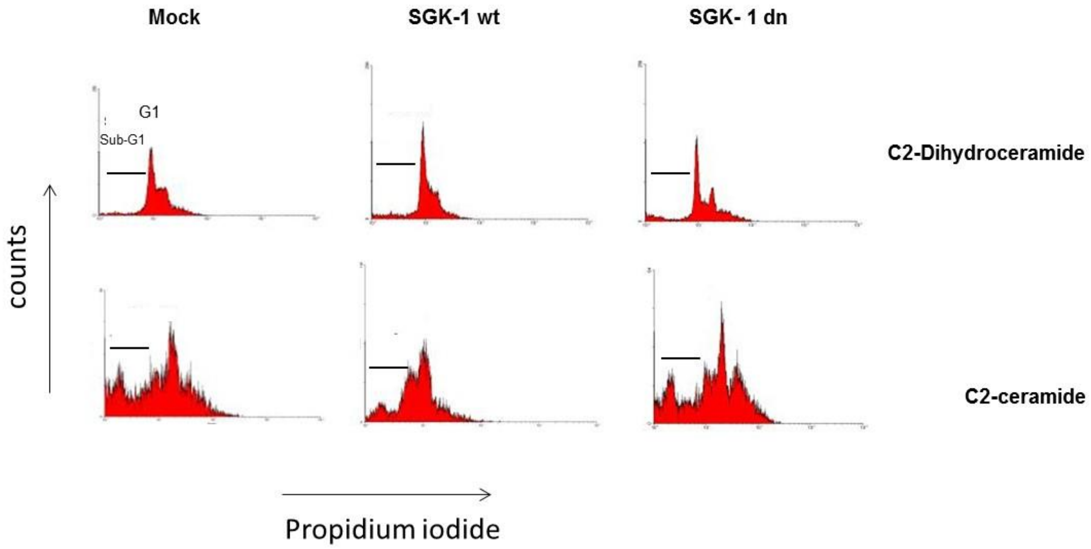

Supplementary Figure S3: Typical flow cytometry experiment in Mock, SGK-1wt and SGK-1dn groups after C_2-Dihy or C_2-ceramide stimulus. Cells were stained with a DNA intercalating dye like

propidium iodide. DNA profile representing cells in G1, S-phase and G2M is reported. Apoptotic cells were present in the sub-G1 population (left to the G1 peak).

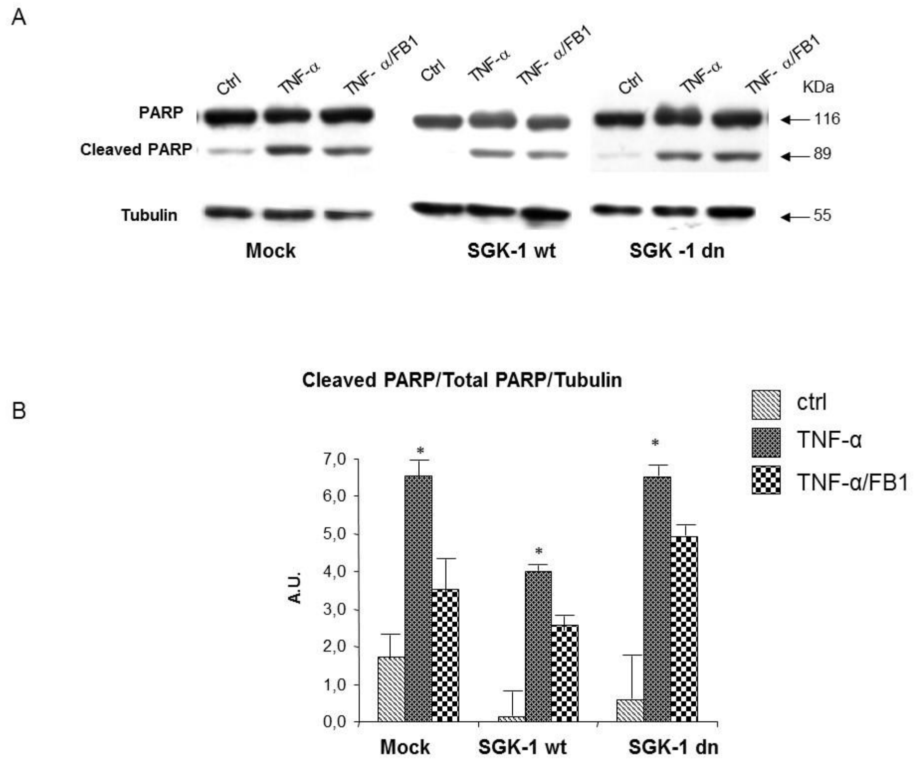

Supplementary Figure S4: HEK-293 cells were transfected with Mock, SGK-1 wt and SGK-1 dn constructs. Cells were treated with TNF-α (100 ng/ml) for 72h in presence or not of 100µM FB1 (Fumonisin-B1) to inhibit ceramide production. After 72h of treatment with TNF-α, cells were immunoblotted with specific antibodies for full-length (116 KDa) and cleaved PARP-1 (89 KDa). Tubulin was used as a loading control. Blots shown are representative of three independent experiments (**Panel A**). A quantification of three independent experiments by scanning densitometry is shown (**Panel B**), *$p<0.05$ TNF-α (Mock, SGK-1 wt and SGK-1 dn) vs TNF-α/FB1 (Mock, SGK-1 wt and SGK-1 dn), *$p<0.05$ TNF-α (SGK-1 wt) vs. TNF-α (Mock and SGK-1 dn), N=3. Results are expressed as means +/- SD.

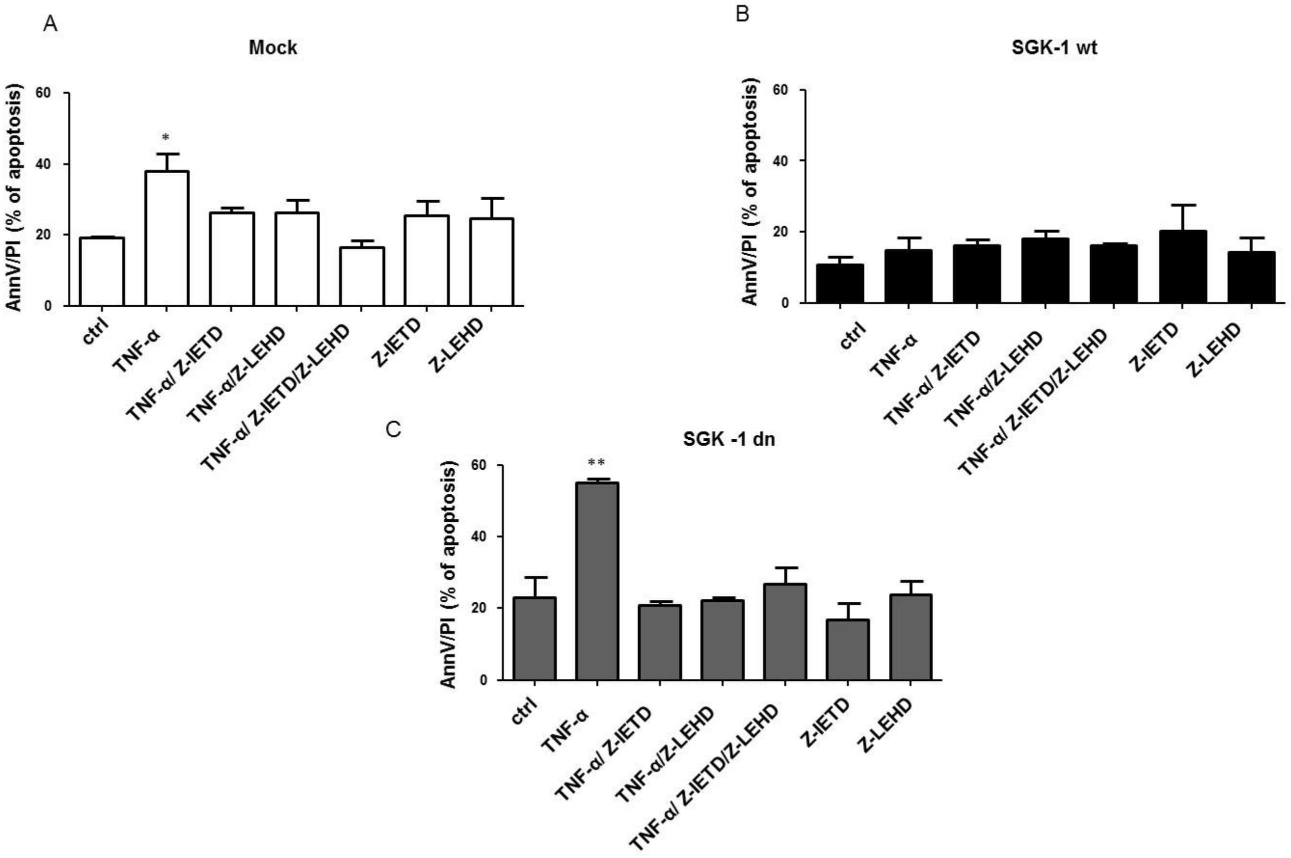

Supplementary Figure S5: HEK-293 cells were transfected with Mock, SGK-1wt and SGK-1dn constructs and stimulated with caspases-8 inhibitor Z-IETD-fmk, (20μM), and/or caspases-9 inhibitor, Z-LEHD-fmk, (20μM), 60 min before 72h of TNF-α treatment (100ng/ml). Apoptotic cells were determined by annexin V-FITC/PI method. These results were obtained from at least 3 different experiments, *$p<0.05$ TNF-α vs. ctrl, TNF-α/Z-IETD, TNF-α/Z-LEHD, TNF-α/Z-IETD/Z-LEHD (Mock). **$p<0.01$ TNF-α vs. ctrl, TNF-α/Z-IETD, TNF-α/Z-LEHD, TNF-α/Z-IETD/Z-LEHD, Z-IETD, Z-LEHD (SGK-1dn); N=3. Results are expressed as means +/- SD.
